# Concentration dependent energy levels shifts in donor-acceptor mixtures due to intermolecular electrostatic interaction

**DOI:** 10.1038/s41598-019-48877-9

**Published:** 2019-08-27

**Authors:** Saientan Bag, Pascal Friederich, Ivan Kondov, Wolfgang Wenzel

**Affiliations:** 1 Center for Condensed Matter Theory, Department of Physics, Indian Institute of Science (IISc), Bangalore, India; 2 Steinbuch Centre for Computing (SCC), Karlsruhe Institute of Technology (KIT), Karlsruhe, Germany; 3 Institute of Nanotechnology (INT), Karlsruhe Institute of Technology (KIT), Karlsruhe, Germany; 4 Department of Chemistry, University of Toronto, Toronto, Canada

**Keywords:** Molecular electronics, Electronic devices

## Abstract

Recent progress in the improvement of organic solar cells lead to a power conversion efficiency to over 16%. One of the key factors for this improvement is a more favorable energy level alignment between donor and acceptor materials, which demonstrates that the properties of interfaces between donor and acceptor regions are of paramount importance. Recent investigations showed a significant dependence of the energy levels of organic semiconductors upon admixture of different materials, but its origin is presently not well understood. Here, we use multiscale simulation protocols to investigate the molecular origin of the mixing induced energy level shifts and show that electrostatic properties, in particular higher-order multipole moments and polarizability determine the strength of the effect. The findings of this study may guide future material-design efforts in order to improve device performance by systematic modification of molecular properties.

## Introduction

Bulk heterojunction solar cells typically contain two or more molecular species, from which at least one material acts as electron donor material and another as electron acceptor, respectively^1–6^. The efficiency of these solar cells depends - among other factors - on exciton separation at interfaces between donor and acceptor materials^7–13^. The separation itself mainly depends on energy level alignment of the local electron affinity (EA) and ionization potential (IP) of donor and acceptor materials^14,15^. Recent work showed that mixing of materials influences the IP as well as the EA of all involved materials^15,16^. The strength of this effect depends on the materials involved in the mixture and on the mixing ratio^15^. Similar effects were discussed in doped organic semiconductors where the electron affinity of dopant molecules changes with doping concentration and choice of host material^17,18^.

During device fabrication, the materials form a phase-separated domain structure with typical domain sizes in the order of several tens of nanometers^2,3,19^. The driving force governing this phase separation is a trade-off between intermolecular interaction energy and mixing entropy of the different materials. At finite temperatures, entropic contributions hinder full phase separation, which means that both phases of the bulk heterojunction consist of material mixtures with mixing ratios close to, but not equal to one (or zero, respectively)^6^. Presently little is known about the detailed nature of the chemical composition of the interfaces in hetero-junctions, but studies have shown large effects of interface structure on charge separation^15,20^. Considering these two observations (mixture-dependent energy level shifts and partially mixed phases in bulk heterojunctions), it becomes clear that understanding and control of material composition at interfaces has a strong influence on the energy level alignment, exciton separation and other processes, which play a crucial role in device performance^20^.

Computational models provide a unique opportunity to unravel the relevance and relative importance of specific factors, such as polarizability and multipole moment on the energy levels. While such models are widely used to predict properties of single isolated molecules they still struggle with the accurate prediction of bulk properties of ordered and disordered materials or material interfaces. QM/MM models were used to quantify systematic energy level difference between isolated molecules in vacuum and bulk materials^21–25^, while other studies focused on the energy level alignment in mixed materials and crystalline interfaces^15,20^. In some scenarios the polarizability of molecules was shown to be the main reason for energy level differences between molecules in vacuum and bulk materials^22,23^ while electrostatic properties such as quadrupole moments dominate energy level shifts at ordered interfaces^20^.

This study systematically analyses the dependence of ionization energies of molecules in amorphous organic materials on their microscopic environment. We analyze mixtures of the donor materials rubrene, NPB, bDIP and 6T with the acceptor molecule C_60_ using a combination of molecular simulation^26–28^ and electronic structure methods^29,30^. Experimental measurements by Graham *et al*. show that the donor IP of bDIP and 6T increases with increasing C_60_ content, while the IP of rubrene and NPB remain relatively constant^16^. At the same time, 6T and bDIP influence (decrease) the EA of the acceptor molecule C_60_ when their concentration is increased. Here, we investigate the role of local electrostatic interaction (in terms of polarity and polarizability) between acceptor and donor molecules to understand the molecular origin of the concentration dependent energy level shifts. We show that in contrast to the assumptions by Graham *et al*.^16^, the electrostatic properties (multipole moments) of the molecules play a stronger role than their polarizability. In particular, we show that the net electrostatic potential caused by molecules at the positions of their direct neighbors significantly differs between different materials and is responsible for the energy level shifts in material mixtures.

## Methods

**Figure 1:**
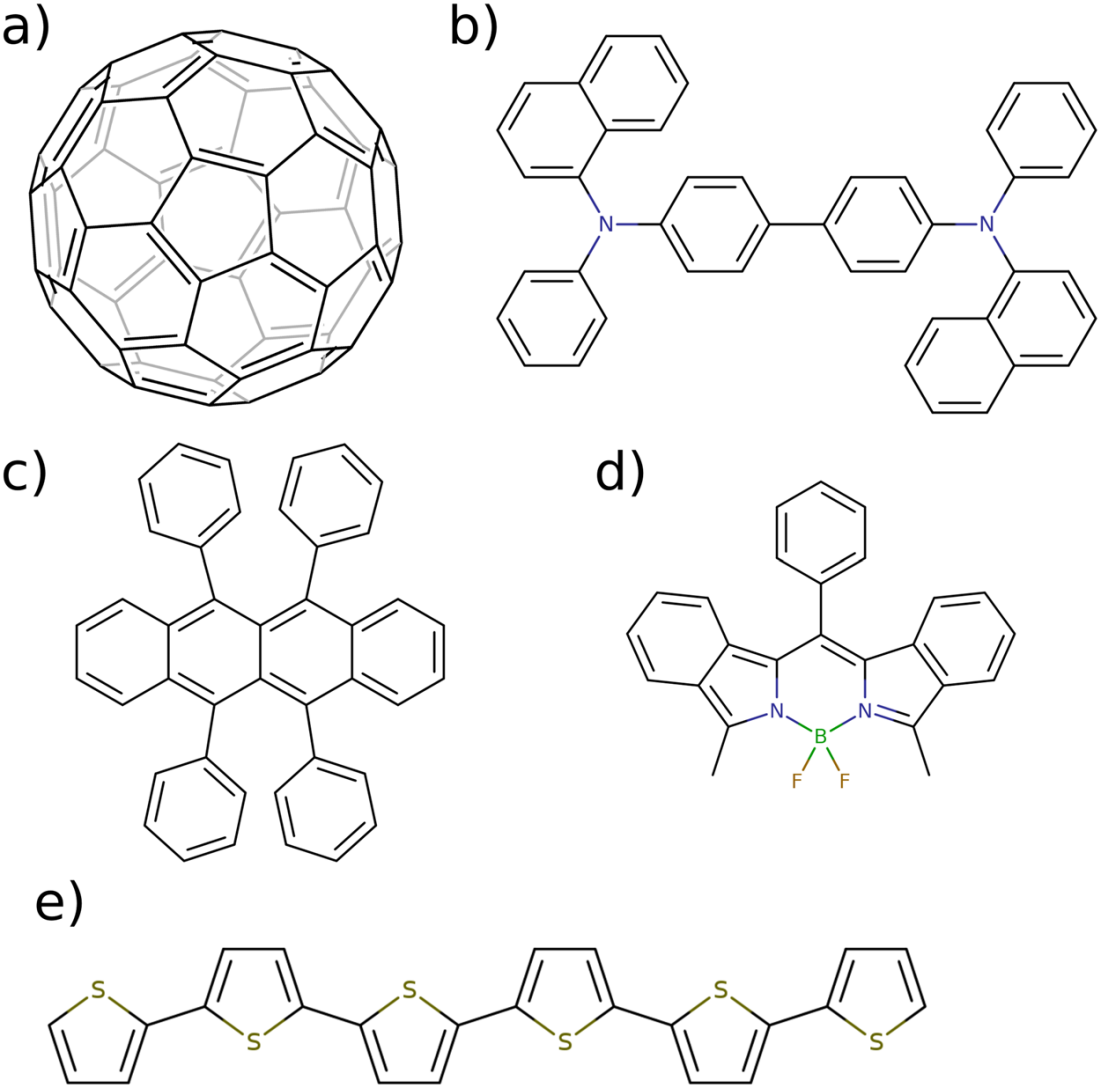
Molecular structures of (**a**) Acceptor molecule C60, (**b–e**) Donor materials NPB, rubrene, bDIP and 6T.

In order to calculate the ionization energy distribution of mixed amorphous organic materials we first generate atomistically resolved morphologies of the relevant systems. In this work, these are obtained using a Metropolis Monte Carlo based simulated annealing protocol^26^. In this approach, molecules are sequentially deposited in a simulation box using a simulated annealing protocol where the temperature is cooled down from 4000 K to 300 K in 140,000 Metropolis Monte Carlo steps. The energy of the molecules is evaluated using an intramolecular force field describing dihedral rotations and Lennard-Jones interaction as well as an intermolecular force field for electrostatic interaction and Lennard-Jones interaction. The molecule specific intramolecular dihedral force field is obtained using a PM6^31,32^ based protocol in which all dihedral angles are rotated independent from each other in a sequence of steps. In each step, one dihedral angle is fixed at a given position while the rest of the molecule is relaxed using a semi-empirical PM6 geometry optimization. The dihedral force field is obtained using spline-fits to the total energy curves of all dihedral rotations. Using this protocol, we generated atomistic structures of mixtures of C_60_ with the donor molecules shown in Fig. 1b–e. We used C_60_ concentrations of 25 wt.%, 50 wt.%, 75 wt.%, 90 wt.% and 100 wt.%. Each morphology contained approximately 1200 molecules.

IP and EA levels were computed using the Quantum Patch method^29,30^. Here, the total energies of all molecules in the central region of a morphology are calculated in the charged and neutral state using hybrid density functional theory (B3-LYP^33^, def2-SV(P)^34^). IP/EA levels are then calculated as total energy differences between the positively/negatively charged states of molecules and their neutral state. Polarization effects and intermolecular electrostatic interaction are explicitly included by self-consistent evaluation of the system of electrostatically coupled molecules. This allows simultaneous localization of additional charges on given molecules in the systems and evaluation of polarization effects in large clusters of the system.

Depending on the concentration of host and guest molecules, the number of molecules (per type) evaluated in each morphology ranged from 11–200 (numbers smaller than 47 only occurred for donor molecules in the 90 wt.% C_60_ morphologies). The Quantum Patch method furthermore allows us to extract IP/EA level distributions as well as HOMO/LUMO energy distribution with and without polarization effects of the environment. This helps us to separate between energy level shifts due to the electrostatic properties of the surrounding molecules and polarization effects due to different polarizabilities of the surrounding molecules.

## Results

The Quantum Patch calculations enable us to investigate the electrostatic interaction between the molecules in the amorphous mixtures. The electrostatic interaction can be divided into two contributions, first being the electrostatic moments of the charge distribution of the molecules and second being polarization effects due to creation of positive polarons during the IP measurements/calculations. To distinguish between the two effects, we extract IP and HOMO distributions before and after the self-consistent analysis of the polarization effects. It is known from previous theoretical studies (see *e.g*. de Silva *et al*.^23^) that the IP shift when going from vacuum to solid state can be attributed to polarization effects, while the broadening of the IP distribution is mainly caused be the electrostatic moments of the disordered molecules. Further studies of doped organic semiconductors show, that the electron affinity of the dopant molecules depends on the host material as well as on the ratio between host and dopant^17^. In this work, we study the effect of varying type and composition of the molecular environment and its influence on the IP and HOMO level position in mixed donor-acceptor systems.

**Figure 2:**
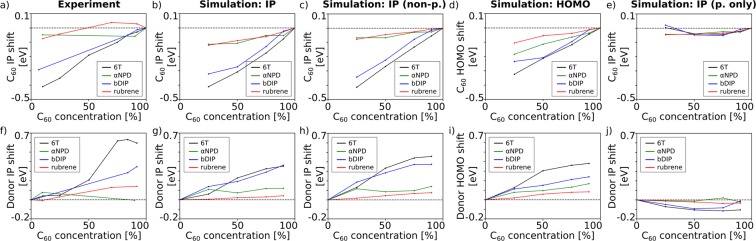
Shift of the IP of C_60_ (**a–e**) and donor materials (**f–j**) as a function of the C_60_ content. Experimental data by Graham *et al*. is shown in (**a,f**). Calculated IP shifts are shown in (**b,g**). Panels (c,h) show IP shifts without the influence of polarization effects and panels (d,i) show calculated HOMO energy shifts instead of IP shifts. Panels (e,j) show the energy level shifts as a function of pure polaronic polarization (dynamic polarization), which we calculated as the difference of panels (b/c) and (g/h), respectively.

The results of the Quantum Patch calculations as well as a comparison with experimental data by Graham *et al*. are shown in Fig. 2. In all cases, the IP of C_60_ decreases when decreasing the C_60_ content of the samples. Mixing C_60_ with rubrene or αNPD leads to smaller shifts of the C_60_ IP than mixing it with the 6T oligomer or bDIP. A maximum shift of approximately −0.4 eV is observed at 90% 6T content (see Fig. 2a). The calculated IP shifts shown in Fig. 2b confirm the experimental data and thus offer the possibility to investigate the microscopic origin of the effect.

In Fig. 2c, we again show the dependence of the IP of C_60_ as a function of the C_60_ concentration, but this time, we extracted the results before evaluating the polarization between molecules. This corresponds to a model where only the electrostatic moments of neighboring molecules are considered when evaluating the IP distributions of the various mixtures. We find that the non-polarized calculations of the IP energy as a function of the C_60_ content show the same trend as the experimental data as well as the simulations including polarization effects, which leads to the conclusion that differences in the electrostatic properties of the donor molecules lead to the observed energy shifts rather than differences in polarizability of the molecules. In a third test (see Fig. 2d), we only extracted the HOMO energies of the molecules rather than the IP values. The main difference between the calculation of HOMO and IP energies is that the IP calculation requires to explicitly charge the molecule (one at a time) and evaluate their total energy including polarization effects of the environment. The polarizability of the environment therefore plays a more important role in the IP compared to the HOMO energy. We find the same trend as in the IP calculations with and without polarization effects described before, which again indicates that the experimentally observed IP shifts can be attributed to differences between the electrostatic moments of the donor molecules rather than to differences in their polarizability.

Varying the C_60_ content of the samples not only shifted the IP of C_60_ but also that of the donor molecules themselves. This is shown in Fig. 2f–i, where again panel (f) shows the experimental data by Graham *et al*., panels (g) and (h) show the calculated IP values with and without polarization and panel (i) shows the HOMO energy as a function of the C_60_ content. Increasing the C_60_ content again leads to strong IP shifts of up to 0.6 eV in case of 6T and bDIP mixtures and to weaker IP shifts (0.1–0.2 eV) in case of αNPD and rubrene. All simulated curves show the same trends (6T > bDIP > αNPD > rubrene) and agree well with the experimental data, apart from a slight underestimation of the IP shift of 6T. We therefore conclude again, that polarization effects do not seem to play a major role as we observe the same behavior when artificially switching off polarization effects (Fig. 2h) or only analyzing the HOMO energies of neutral molecules (Fig. 2i). Figure 2e,j show the energy level shifts as a function of pure polaronic polarization (dynamic polarization), which we calculated as the difference of panels (b/c) and (g/h), respectively. It is visible that the dynamic polarization only weakly depends on the environment and is thus not responsible for the energy level shift as a function of the mixing ratio.

**Figure 3:**
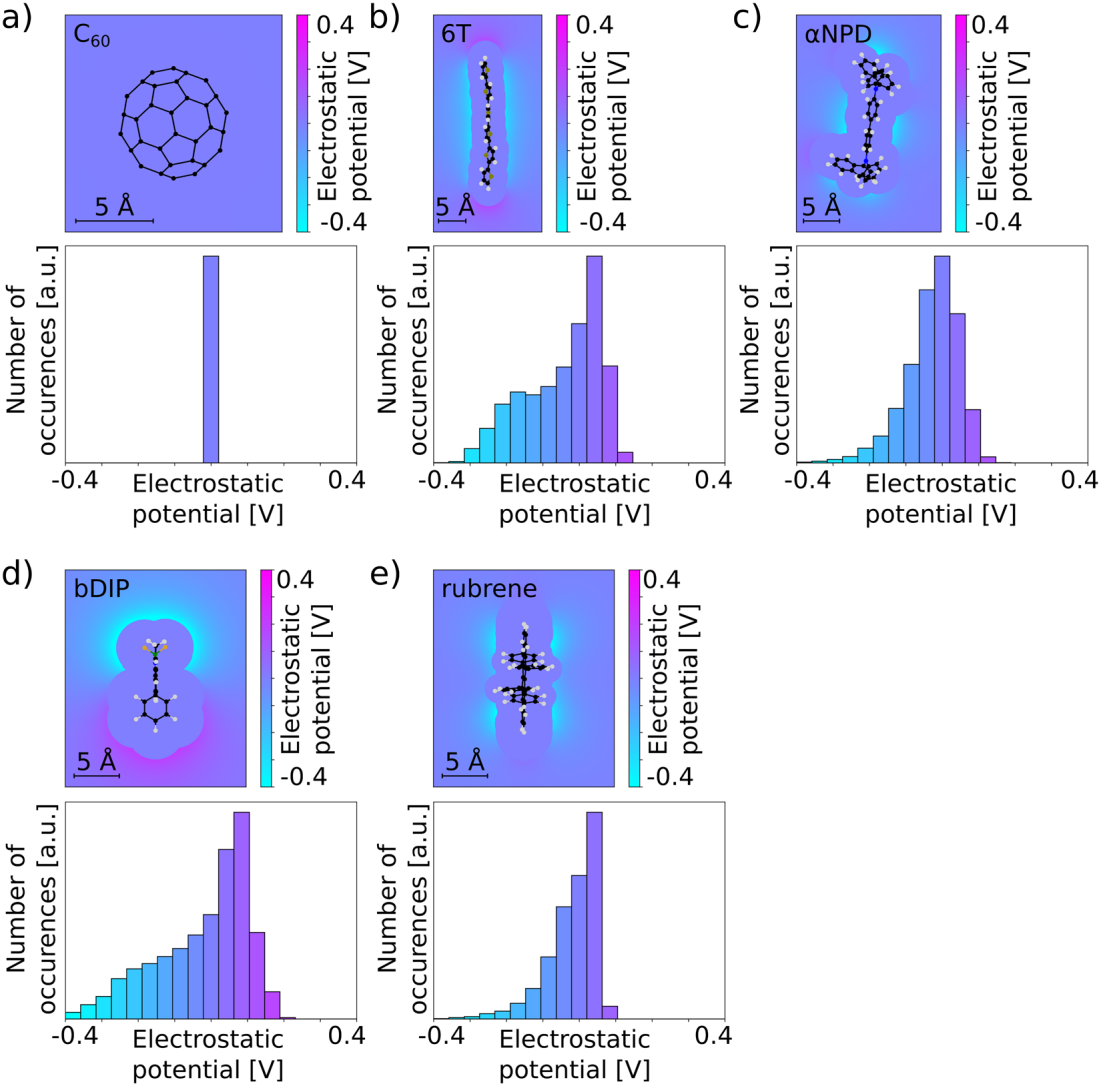
The panels show the electrostatic potential caused by the charge density of the molecules as well as histograms of the electrostatic potential. The planes on which the electrostatic potential is plotted include the points of highest potential. The histogram includes points in distances between 3.2 Å and 4.6 Å.

To further investigate the origin of the energy level shift and to understand the differences between the different donor:C_60_ mixtures, we analyzed the electrostatic potential created by the charge density of all molecules. In Fig. 3, we show the electrostatic potential on planes through the center of each molecule and through the spatial region with highest electrostatic potential. We find that the highly symmetric C_60_ molecule causes almost no electrostatic potential while the less symmetric donor molecules show regions of negative as well as positive electrostatic potential. In Friederich *et al*.^35^, we have shown that the electrostatic potential of molecules in spatial regions between 3.2 Å and 4.6 Å around the molecules strongly correlates with the electrostatic interaction between the molecules. We therefore analyzed the electrostatic potential distribution on points between these distances to the molecule. The corresponding histograms in Fig. 3 show an asymmetry of the distributions with large shoulders of high negative electrostatic potential especially in case of 6T and bDIP, which can be responsible for the IP/HOMO shifts shown in Fig. 2. The regions of high negative potential are mainly caused by electron rich groups such as nitrile or fluorine, as well as the π-orbitals of the thiophene backbone of 6T and the BF_2_ group of bDIP. Electrostatic interaction between such groups and the orbitals/electrons of neighboring molecules shifts up the orbital energies and leads to smaller ionization energies.

**Figure 4:**
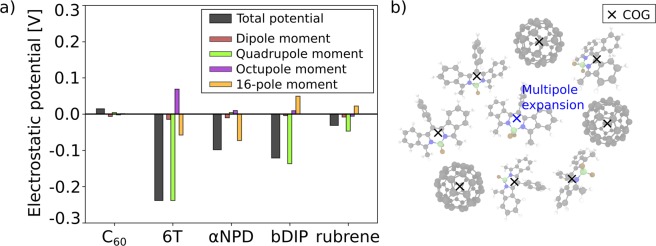
(**a**) Sum of the electrostatic potential created by the (guest) molecules at the center-of-geometry (COG) positions of the thirty nearest neighbors (see panel b)). Albeit having no net charge (monopole moment), the guest molecules (in particular 6T and bDIP) create a negative electrostatic potential which causes the shift of the energy levels of both guest and host (C_60_) molecules as observed in experiment and simulations (see Fig. 2). A multipole analysis identifies the electrostatic moments responsible for the energy level shift.

To analyze this effect in a more quantitative way, we calculated the net (summed up) electrostatic potential caused by the (donor) molecules at center-of-geometry (COG) positions of the neighboring twenty molecules. The results of this analysis are shown in Fig. 4. In principle, the electrostatic potential caused by any multipole moment is symmetric so if the environment is symmetric, one expects a net electrostatic potential of zero. The dipole contribution spatially varies very slowly, which is why its contribution is almost zero (see red bars in Fig. 4). Higher order moments are responsible for spatially stronger varying potentials where the shape of the molecule and its neighbors matters. This systematic inhomogeneity of the local environment leads to non-zero net potentials, which systematically shift the energy levels of neighboring molecules.

In case of 6T and bDIP, the net electrostatic potential exceeds −0.1 eV. An expansion of the electrostatic potentials caused by the molecules into their multipoles reveals that in case of 6T and bDIP, the quadrupole moments are the main source of the negative electrostatic potential. In case of αNPD, all moments up to the octupole moment do not cause a net electrostatic potential making the 16-pole moment the source of the (small) energy level shift. In case of C60 and rubrene, no significant multipole moments are visible, which corresponds to negligible energy level shifts caused by these materials.

## Summary and Conclusion

We have shown in this work that multiscale simulations can quantitatively predict the shifts of energy levels such as the ionization potential of a materials as a function of the material mixture, an effect that was experimentally observed by Graham *et al*.^16^. Our calculations indicate that the experimentally observed IP level shift due to mixing of C_60_ with different donor materials is caused by the spatially inhomogeneous electrostatic potentials of in particular sexithiophene (6T) and bDIP rather than by differences of the polarizabilities of the donor molecules. This finding has important consequences for the design of mixtures with a desired EA/IP, because the electrostatic potential of a molecule is more easily modulated by adding functional groups than by changing the polarizability. We find that electrostatic interaction between electronegative groups of the donor molecules and the electrons of neighboring molecules shifts up the HOMO energies of the surrounding material and reduces its IP. As the electrostatic potential caused by C_60_ is negligibly small, the IP of both C_60_ and the donor material only depend on the C_60_ concentration and the electrostatic potential of the donor molecule. The findings in this work confirm experimental observations^16^ that the IP of a material is not a pure material property but also depends on the chemical environment of a molecule in particular in material mixtures with a given mixing ratio.

Energy level alignment in bulk heterojunction organic solar cells influences the separation of excitons and thus determines the device performance. Control of the energy levels, in particular of the ionization potential of the electron donor and the electron affinity of the electron acceptor materials plays a crucial role in systematic efforts to understand and improve the quantum efficiency and the power conversion efficiency. According to the findings of this work, the addition of functional groups (both nonpolar or polar) with characteristic electrostatic properties therefore not only changes the intrinsic energy levels of the molecules but also influences the energy levels of neighboring molecules. This effect has to be considered when designing new organic solar cells. The results presented here might help future efforts in material design to systematically exploit this effect and to further improve the efficiency of organic solar cells.
